# Adverse Childhood Experiences and Electronic Cigarette Use among U.S. Young Adults

**DOI:** 10.3390/toxics11110907

**Published:** 2023-11-06

**Authors:** Afolakemi C. Olaniyan, Laura A. Nabors, Keith A. King, Ashley L. Merianos

**Affiliations:** 1 School of Population & Health Sciences, Dillard University, New Orleans, LA 70122, USA; olaniyai@mail.uc.edu; 2 School of Human Services, University of Cincinnati, Cincinnati, OH 45221, USA

**Keywords:** electronic nicotine delivery systems, vaping, adverse childhood experiences, young adult

## Abstract

(1) Background: Adverse childhood experiences (ACEs), which are potentially traumatic childhood events, have been associated with increased tobacco product use. Less is known about electronic cigarette (e-cigarette) use during young adulthood. This study explored the associations between ACEs and current e-cigarette use among U.S. young adults. (2) Methods: This study was a secondary analysis of 2021 Behavioral Risk Factor Surveillance System data including 2537 young adults aged 18–24 years. Unadjusted and adjusted logistic regression analyses were conducted. (3) Results: Of the participants, 19.2% currently used e-cigarettes, and 22.1% reported 1 ACE, 13.0% reported 2 ACEs, 10.7% reported 3 ACEs, and 30.6% reported ≥4 ACEs. Unadjusted results indicated that participants who experienced 1 ACE (odds ratio (OR) = 1.76, 95% confidence interval (CI) = 1.01–3.07), 2 ACEs (OR = 2.18, 95%CI = 1.24–3.83), 3 ACEs (OR = 2.63, 95%CI = 1.41–4.90), and ≥4 ACEs (OR = 3.69, 95%CI = 2.23–6.09) were at increased odds of reporting current e-cigarette use than participants who experienced 0 ACEs. Adjusted results indicated that participants who experienced 3 ACEs were at 2.20 times higher odds (95%CI = 1.15–4.23) and participants who experienced ≥4 ACEs were at 2.73 times higher odds (95%CI = 1.58–4.71) of reporting current e-cigarette use than participants who experienced 0 ACEs. (4) Conclusions: Young adults exposed to ACEs are at risk of using e-cigarettes.

## 1. Introduction

Electronic cigarette (e-cigarette) use is a major public health problem among young adults in the United States (U.S.) [1]. Previous research on current e-cigarette use indicated that U.S. young adults aged 18–24 years experienced the greatest increase in current use patterns [2,3]. Even though the recent trends using 2017–2020 data indicated that current e-cigarette use has decreased among young adults aged 18–20 years, daily use has increased among young adults aged 21–24 years [4]. In 2021, 11% of the U.S. 18–24-year-old age group reported using e-cigarettes every day or on some days compared to a 4% use prevalence for the overall U.S. adult population [5]. Further, another study discovered that young adults usually start using e-cigarettes at a higher rate than adolescents [6].

Several reasons have been noted for the growth in e-cigarette use among young adults, including lower perceived harm associated with e-cigarette use compared to combustible cigarette smoking [7]. Although e-cigarettes do not involve combustion, ingredients can include solvents, flavorings, and nicotine also found in combustible cigarettes [8,9]. Beyond the risk of addiction to nicotine, this ingredient is harmful to brain development and can cause neurological effects [10]. Rising evidence suggests that e-cigarettes can be toxic and can cause negative neurodevelopmental, pulmonary, cardiovascular, and immunologic effects on young adults’ health [11]. Another review indicated that e-cigarettes contain numerous potential toxicants including flavorings that may irritate the lungs causing chronic inflammation [12]. Additionally, a systematic review of the literature found that e-cigarette use is associated with increased mental health issues among young adults [13].

Adverse childhood experiences (ACEs) provide a framework that encompasses different types of abuse (i.e., physical, emotional and psychological) and household dysfunction (i.e., mental illness, substance abuse, incarceration, and physical violence among household members) experienced during childhood and adolescence [14]. ACEs are another public health burden among U.S. adults with over 60% experiencing at least 1 ACE in their lifetime, and 25% having experienced 3 or more ACEs [15]. Studies have linked ACEs to physical health consequences including risk of having increased blood pressure during young adulthood [16], as well as future cardiovascular disease development [17,18,19]. Other negative health outcomes associated with ACEs among adults include myocardial infection, coronary heart disease, stroke, diabetes, and asthma [20]. These negative health consequences during adulthood may originate from ACEs, which cause toxic stress or the prolonged activation of the stress-response system that can interrupt children’s immune systems, nervous systems, and can even have epigenetic effects such as DNA alterations [21]. These physiological changes can impact their executive functions, including, but not limited to, their decision-making processes, impulse control, behavioral self-regulation, and mood [22]. This can place them at high risk for engaging in health-risk behaviors like tobacco use (e.g., combustible cigarettes, smokeless tobacco products) during adolescence [23] and early adulthood [24,25,26].

Limited research has assessed the association between ACEs and current e-cigarette use during young adulthood, particularly producing results that are generalizable to young adults across the U.S. For example, prior research has used a smaller, community sample of young adults aged 18–21 years to assess lifetime use versus current use, and found that those who experienced maltreatment during their childhood were more likely to engage in e-cigarette use during their lifetime [27]. Similarly, a smaller, community-based study among young adults aged 18–25 years found that compared to those with low ACEs, those with a higher number of cumulative ACEs reported current cigarette smoking, but did not assess e-cigarette use [28]. Moreover, the majority of the literature has concentrated on younger populations. These studies have shown that ACEs are predictors of e-cigarette initiation among middle school students [29], and a higher cumulative number of ACEs (i.e., ≥4) is associated with using e-cigarettes among high school and vocational school students [30]. Other research has examined ACEs as predictors of e-cigarette use among a wide age range of adults living in one U.S. state, Florida, and found that a higher cumulative number of ACEs (i.e., ≥4) is associated with current e-cigarette use [31]. Another study assessed the potential effect of sex on the association of ACEs and current e-cigarette and cigarette smoking patterns among U.S. adults of all ages, and reported that there was no interaction by sex, but a higher cumulative number of ACEs (i.e., ≥4) was associated with use [32]. More research is needed to assess this association among a national sample of young adults living across the U.S.

This study aimed to fill gaps in the literature and provide professionals with information that can be used to tailor e-cigarette prevention and cessation programming to U.S. young adults who may have experienced ACEs. Therefore, the primary objective of this study was to examine the associations between ACEs and current e-cigarette use among U.S. young adults. We hypothesized that U.S. young adults with a cumulative higher number of ACEs would be at increased odds of reporting current e-cigarette use compared to U.S. young adults with 0 ACEs. We also hypothesized that when compared to 0 ACEs, U.S. young adults who experienced individual ACEs (e.g., emotional abuse, physical abuse) would be at increased odds of reporting current e-cigarette use.

## 2. Materials and Methods

### 2.1. Participants and Procedures

This study was a secondary analysis of 2021 Behavioral Risk Factor Surveillance System (BRFSS) data [33], and included 2537 young adults aged 18–24 years with data available on ACEs and current e-cigarette use. Specifically, of the 26,025 young adults aged 18–24 years who participated in the survey, 2556 had ACE data available for analysis. Of those, 2537 had current e-cigarette use and covariate data available and were included in the current study’s analysis.

The BRFSS is a behavioral health-based telephone survey administered by the Centers for Disease Control and Prevention (CDC), which is conducted using a computer-assisted telephone interview system among adults residing in the U.S. [34]. Specifically, the original goal of the BRFSS is to collect uniform data from all 50 U.S. states including the District of Columbia, Guam, the U.S. Virgin Islands, and Puerto Rico about health-risk behaviors, chronic diseases, healthcare access, and preventive health services utilization associated with the leading causes of U.S. morbidity and mortality [34]. The 2021 BRFSS median response rate for cellular and landline telephones was 44% [35].

A university-based Institutional Review Board (IRB) approved this study with a “not human subjects research determination” based on using public, de-identified BRFSS data.

### 2.2. Measures

#### 2.2.1. Independent Variables: ACEs

The main independent variables of interest were about ACEs, which was assessed in the “Adverse Childhood Experiences” section of the 2021 BRFSS by asking participants the following no/yes questions: “Now, looking back before you were 18 years of age…” (1) “Did you live with anyone who was depressed, mentally ill, or suicidal?”, (2) “Did you live with anyone who was a problem drinker or alcoholic?”, (3) “Did you live with anyone who used illegal street drugs or who abused prescription medications?”, (4) “Did you live with anyone who served time or was sentenced to serve time in a prison, jail, or other correctional facility?”, (5) “Were your parents separated or divorced?”, (6) “How often did your parents or adults in your home ever slap, hit, kick, punch or beat each other up?”, (7) “Not including spanking, (before age 18), how often did a parent or adult in your home ever hit, beat, kick, or physically hurt you in any way?”, (8) “How often did a parent or adult in your home ever swear at you, insult you, or put you down?”, (9) “How often did anyone at least 5 years older than you or an adult, ever touch you sexually?”, (10) “How often did anyone at least 5 years older than you or an adult, try to make you touch them sexually?”, and (11) “How often did anyone at least 5 years older than you or an adult, force you to have sex?” [36]. Based on the distributions of the cumulative number of ACEs (range 0–11), we followed prior work by the CDC [37], and assessed the number of ACEs categorically: 1, 2, 3, and ≥4 ACEs. For the assessment of individual ACE types, we assessed each ACE individually, with the exception of the 3 ACEs about sexual abuse. If participants answered “yes” to at least 1 of the 3 sexual abuse-related ACE questions about someone touching them sexually, making them touch someone sexually, or someone forcing them to have sex, then they were considered to have experienced the ACE of sexual abuse.

#### 2.2.2. Dependent Variable: Current E-Cigarette Use

Current e-cigarette use was the dependent variable of interest for this study. E-cigarette use was assessed in the “Tobacco Use” core section of the 2021 BRFSS by asking participants one question about their past 30-day use: “Do you now use e-cigarettes or other electronic vaping products every day, some days or not at all?” [36]. Based on the distribution of the original responses, we used the calculated variable provided in the 2021 BRFSS data set that combined the options of “every day” (*n* = 241) and “some days” (*n* = 261) to define current e-cigarette use. 

#### 2.2.3. Covariates: Participant Characteristics

We included the following sociodemographic covariates: young adult sex, race/ethnicity, education level, and income level. Young adult race/ethnicity included the categories of non-Hispanic White, non-Hispanic Black, non-Hispanic Other/Multiracial, and Hispanic. Education level included the categories of high school graduate or less, some college, and college graduate. Income level included the categories of <USD 25,000, USD 25,000-USD 49,999, ≥USD 50,000, and unspecified (i.e., unknown, not sure). Since current use of two or more tobacco products is prevalent among U.S. young adults [5], we also included the covariate of current other tobacco use (i.e., combustible cigarettes, smokeless tobacco).

### 2.3. Data Analysis

All data analyses were conducted using the SPSS Complex Samples (version 28.0). We used the BRFSS-provided sampling weights to reflect the U.S. population of young adults aged 18–24 years [38]. To describe the study population and determine the extent of current e-cigarette use, we calculated descriptive statistics and unweighted sample counts and weighted column percentages. We conducted chi-square tests to examine the associations between participant characteristics and current e-cigarette use. We also reported descriptive statistics of participant characteristics by cumulative number of ACEs, and present unweighted sample counts and weighted column percentages.

To answer our main study objective, we conducted an unadjusted logistic regression analysis to assess the association between cumulative number of ACEs and current e-cigarette use, and present the odds ratios (ORs), 95% confidence intervals (CIs) and *p*-values. We then conducted an adjusted logistic regression analysis to assess this relationship while adjusting for the covariates (i.e., young adult sex, race/ethnicity, education level, income level, and current other tobacco use), and present the adjusted ORs (AORs), 95%CIs, and *p*-values.

As a follow-up analysis, we built nine separate unadjusted logistic regression models and nine separate adjusted logistic regression models to assess the associations between each individual ACE type and current e-cigarette use, and present either ORs or AORs, 95%CIs, and *p*-values. We used *p* < 0.05 to indicate statistical significance for all analyses and reduce the probability of committing a Type I error.

## 3. Results

A total of 44.6% of participants were female, and 66.8% were non-Hispanic White, 12.9% were Hispanic, 11.0% were non-Hispanic Black, and 9.3% were non-Hispanic Other/Multiracial (Table 1). Education level varied with 44.6% of participants obtaining a high school degree or less, 35.8% obtaining some level of college education, and 19.6% obtaining a college degree at the time of survey completion. A total of 14.6% of participants had an income level <USD 25,000, 24.3% had an income level USD 25,000–USD 49,000, and 35.3% had an income level ≥USD 50,000. Nearly 9% of participants reported current other tobacco use (i.e., combustible cigarettes, smokeless tobacco products). A total of 19.2% (*n* = 502) of participants currently used e-cigarettes. Concerning the cumulative number of ACEs, 23.6% (*n* = 602) of participants reported 0 ACEs, 22.1% (*n* = 543) reported 1 ACE, 13.0% (*n* = 378) reported 2 ACEs, 10.7% (*n* = 228) reported 3 ACEs, and 30.6% (*n* = 786) reported ≥4 ACEs.

### 3.1. Participant Characteristics Based on Current E-Cigarette Use

Participants’ sex, race/ethnicity, education level, and current other tobacco use differed based on current e-cigarette use (see Table 1). Participants who were male (64.9%), non-Hispanic White (75.4%), had an education level of a high school degree or less (50.8%), and engaged in current other tobacco use (23.5%) had high reports of current e-cigarette use.

### 3.2. Participant Characteristics Based on Cumulative Number of ACEs

Table 2 presents the participant characteristics based on the cumulative number of ACEs. As the number of ACEs increased from 0 ACEs to ≥4 ACEs, the percentages of current e-cigarette use and current other tobacco use increased. Male participants reported a higher percentage (56.2%) of experiencing 0 ACEs than female participants (43.8%), but male and female participants had similar percentages of experiencing ≥4 ACEs (49.8% and 50.2%, respectively). Participants’ race/ethnicity, education level, and income level had varying percentages across the cumulative number of ACEs (see Table 2).

### 3.3. Current E-Cigarette Use and Cumulative Number of ACEs

Unadjusted logistic regression model results indicated that participants who experienced 1 ACE (OR = 1.76, 95%CI = 1.01–3.07, *p* = 0.046), 2 ACEs (OR = 2.18, 95%CI = 1.24–3.83, *p* = 0.007), 3 ACEs (OR = 2.63, 95%CI = 1.41–4.90, *p* = 0.002), and ≥4 ACEs (OR = 3.69, 95%CI = 2.23–6.09, *p* < 0.001) were at increased odds of reporting current e-cigarette use compared to participants who experienced 0 ACEs (Table 3).

Adjusted logistic regression model results indicated that participants who experienced 3 ACEs were at 2.20 times higher odds (95%CI = 1.15–4.23, *p* = 0.018) and participants who experienced ≥4 ACEs were at 2.73 times higher odds (95%CI = 1.58–4.71, *p* < 0.001) of reporting current e-cigarette use compared to participants who experienced 0 ACEs, while adjusting for participants’ sex, race/ethnicity, education level, income level, and current other tobacco use (see Table 3). Concerning significant covariates in the adjusted model, participants who reported current other tobacco use were at 3.61 times higher odds (95%CI = 2.36–5.53, *p* < 0.001) of reporting current e-cigarette use compared to participants who did not report current other tobacco use. Participants who were female (OR = 0.65, 95%CI = 0.48–0.89, *p* = 0.007), non-Hispanic Black (OR = 0.43, 95%CI = 0.25–0.72, *p* = 0.002) or Hispanic (OR = 0.46, 95%CI = 0.26–0.81, *p* = 0.007), and had an education of a college graduate (OR = 0.57, 95%CI = 0.35–0.94, *p* = 0.026) were significantly less likely to report current e-cigarette use compared to participants who were male, non-Hispanic White, and had an education of a high school graduate or less, respectively.

### 3.4. Current E-Cigarette Use and Individual Type of ACEs

The two most frequently reported individual ACE types by participants were experiencing emotional abuse from a parent who swore at them, insulted them, or put them down (48.7%, *n* = 1280) and having parents who divorced or separated (44.5%; *n* = 1071). This was followed by 35.5% (*n* = 927) of participants living with a household member who had a mental illness. Further, a total of 28.0% (*n* = 716) and 18.9% (*n* = 465) of participants lived with a household member who had an alcohol problem and used illicit drugs, respectively. Concerning physical violence-related ACEs, 24.0% (*n* = 631) and 19.3% (*n* = 502) of participants reported being physically abused by a parent and living with household members who engaged in physical violence with each other, respectively. The next prevalent ACEs reported by participants were living with a household member who was incarcerated (17.5%, *n* = 455) and were sexually abused (13.4%, *n* = 343).

Unadjusted and adjusted logistic regression model results indicated that participants who experienced emotional abuse by a parent (OR = 2.14, 95%CI = 1.60–2.87, *p* < 0.001; AOR = 1.83, 95%CI = 1.33–2.51, *p* < 0.001) and lived with a household member who had a mental illness (OR = 1.99, 95%CI = 1.50–2.65, *p* < 0.001; AOR = 1.91, 95%CI = 1.38–2.66, *p* < 0.001) were at higher odds of reporting current e-cigarette use compared to participants who did not experience emotional abuse by a parent or did not live with a household member who had a mental illness, respectively (Table 4).

Unadjusted and adjusted logistic regression model results also indicated that participants who lived with a household member who had an alcohol problem (OR = 2.00, 95%CI = 1.46–2.73, *p* < 0.001; AOR = 1.80, 95%CI = 1.27–2.55, *p* < 0.001) or used illicit drugs (OR = 2.10, 95%CI = 1.52–2.89, *p* < 0.001; AOR = 1.69, 95%CI = 1.19–2.39, *p* = 0.003) were at higher odds of reporting current e-cigarette use compared to participants who did not live with a household member who had an alcohol problem or used illicit drugs, respectively (see Table 4).

Concerning physical violence-related ACEs, unadjusted and adjusted logistic regression model results indicated that participants who experienced physical abuse by a parent (OR = 1.99, 95%CI = 1.46–2.71, *p* < 0.001; AOR = 1.73, 95%CI = 1.21–2.46, *p* = 0.003) and lived with household members who engaged in physical violence with each other (OR = 1.77, 95%CI = 1.30–2.41, *p* < 0.001; AOR = 1.67, 95%CI = 1.18–2.34, *p* = 0.003) were at higher odds of reporting current e-cigarette use compared to participants who did not experience physical abuse by a parent and lived with household members who did not engage in physical violence with each other, respectively (see Table 4).

Unadjusted logistic regression model results indicated that participants who lived with a household member who was incarcerated were at increased odds of reporting current e-cigarette use (OR = 1.62, 95%CI = 1.18–2.23, *p* = 0.003), but the adjusted model including the covariates was not significant. Unadjusted and adjusted model results indicated that participants who experienced sexual abuse (OR = 1.58, 95%CI = 1.09–2.30, *p* = 0.016; AOR = 1.59, 95%CI = 1.01–2.53, *p* = 0.049) were at increased odds of reporting current e-cigarette use compared to participants who did not report sexual abuse (see Table 4).

## 4. Discussion

This study examined the relationship between ACEs and current e-cigarette use among U.S. young adults, and found that a higher cumulative number of ACEs increased the likelihood of reporting current e-cigarette use. Overall, this study reported that about 22% of U.S. young adults experienced 1 ACE, 13% experienced 2 ACEs, 11% experienced 3 ACEs, and 31% experienced ≥4 ACEs. The prevalence of ACEs, with the exception of the current study having double the prevalence of experiencing ≥4 ACEs, was similar to the 2011–2015 BRFSS findings that included adults of all ages and reported about 24% experienced 1 ACE, 13% experienced 2 ACEs, 9% experienced 3 ACEs, and 16% experienced ≥4 ACEs [15]. Additionally, the current study’s results aligned with this prior study by reporting that the top two ACEs experienced by U.S. young adults were emotional abuse from a parent or having parents who separated or divorced, but the current study found a higher prevalence of both emotional abuse from a parent (49% versus 34%) and having parents who separated or divorced (45% versus 28%) [15].

As hypothesized, the unadjusted model results revealed that participants who experienced 1 ACE, 2 ACEs, 3 ACEs, and ≥4 ACEs were at increased odds of reporting current e-cigarette use. However, after sociodemographic covariate adjustment, those with 3 ACEs and ≥4 ACEs remained at increased odds of reporting current e-cigarette use, but no differences were found between 1 ACE and 2 ACEs versus 0 ACEs. Prior research that used 2016 South Carolina BRFSS data also found that experiencing one or more ACEs significantly increased the odds of reporting current e-cigarette use [39]. This study expands on this work by using the recent, national BRFSS data. Further, trends in odds ratios in both the unadjusted and adjusted models demonstrated a possible dose–response relationship in this study. Specifically, as the number of ACEs increased, the odds of engaging in current e-cigarette use also increased. Another study that used 2020 BRFSS data and included a wide range of adults reported similar findings, and also noted that there were varying strengths of associations by participants’ sex with female adults having higher odds of e-cigarette use whereas male adults had higher odds of cigarette smoking and dual e-cigarette and cigarette use [32]. Additionally, an Australian study also suggested a dose–response association between the cumulative number of ACEs and lifetime or past-year e-cigarette use [40]. One potential explanation for this finding is the biologic plausibility of ACEs since related scores can measure the cumulative traumatic stress exposure during childhood or the activated stress response on children’s developing brains [41].

The current study also revealed that most individual ACE types increased the likelihood of reporting current e-cigarette use among U.S. young adults. Specifically, we found that all individual types of ACEs, with the exception of having separated or divorced parents, were associated with current e-cigarette use among young adults. Similar to the current study’s findings, a study that included Canadian adolescents aged 14–17 years reported that experiencing any ACEs increased their odds for current e-cigarette use, and also identified that the individual ACEs of experiencing emotional abuse, household mental health problems and substance abuse placed youth at increased odds of using e-cigarettes [42]. Additionally, a longitudinal study conducted in Canada found that emotional abuse, household mental health problems and substance abuse increased the future risk for vaping from adolescence to young adulthood, providing further support of this study’s findings [43]. Further, another U.S. representative study indicated that physical, emotional, and sexual abuse during childhood was associated with nicotine dependence among adults [44].

The current study also found that about 19% of U.S. young adults engaged in current e-cigarette use in 2021, which was higher than the 11% prevalence reported among 18–24-year-olds in 2021 [5]. Additionally, this study observed that young adults’ sex, race/ethnicity, education level, and current other tobacco use were associated with current e-cigarette use. Specifically, young adults who were male, non-Hispanic White, had a lower education level of high school degree or less, and reported other current tobacco use had a higher prevalence, which is similar to other national findings [5]. E-cigarette use among U.S. young adults should continue to be monitored and efforts are needed to reduce the related sociodemographic disparities.

The strengths of this study are using U.S. representative data that provided a large sample size of U.S. young adults. This secondary research study, however, is not without limitations. First, BRFSS data are cross-sectional in nature and this limited the ability to make causal inferences or assess longitudinal relationships over time. Second, we limited our analysis to young adults with data available on ACEs, current e-cigarette use, and the covariates, and selection bias may have occurred. Additionally, young adults self-reported their responses on the BRFSS, which could have led to recall bias where participants had issues recalling some answers to questions or social desirability bias where participants responded in a socially desirable manner, especially to the sensitive questions about ACEs and current e-cigarette use. Future studies should consider collecting additional data from family members, mental health professionals, and healthcare providers to further assess ACEs in greater detail. Furthermore, this study was unable to assess the frequency of e-cigarette use, such as the number of days of e-cigarette use in the past 30 days, which was not collected in the 2021 BRFSS. We also were unable to assess other potentially important characteristics of e-cigarette use such as device type, brand name, and nicotine content. Future studies should consider collecting detailed data about e-cigarettes and objective measures such as biomarkers related to e-cigarette use including the primary nicotine metabolite of cotinine [45] and biomarkers related to ACEs such as inflammatory markers (e.g., c-reactive protein) [46]. While this study included a specific age group of young adults aged 18–24 years, participants’ age was not publicly available and thus it was not included as a demographic covariate in this study. Finally, we adjusted for other important sociodemographic characteristics and current other tobacco use. We assessed current other tobacco use as a covariate since about 17% and 9% of those who used e-cigarettes also smoked cigarettes or used smokeless tobacco, respectively. However, data were not collected on other tobacco products that should be considered in future studies, such as heat-not-burn tobacco products and cigar products. Additionally, further research should consider assessing ACEs and current tobacco use patterns including exclusive e-cigarette use among U.S. young adults. Future studies should also consider other potential covariates that may influence e-cigarette use that were not included in this study, such as parental vaping or smoking and peer victimization [43].

The study findings demonstrated associations between the cumulative number of ACEs and current e-cigarette use among U.S. young adults. Programs that assist young adults in coping with childhood trauma and maltreatment may contribute to a reduction in their current e-cigarette use. These programs could also provide additional coping resources and increase resilience for those who are currently facing adversity. Studies assessing the impact of messaging to improve parenting and family skills with their youth, targeting reduction in maltreatment, and treating mental health and substance abuse issues for all family members, may mitigate risks for youth later in life such as during young adulthood. These types of studies may inform prevention and intervention programs that aim to promote the healthy functioning of high-risk children, thereby reducing engagement of negative health-risk behaviors such as e-cigarette use during young adulthood.

## Figures and Tables

**Table 1:** Overall participant characteristics and those based on current e-cigarette use among U.S. young adults 18–24 years old, 2021 BRFSS.

		Current E-Cigarette Use		
	Overall (N = 2537)	No Current E-Cigarette Use (*n* = 2035)	Current E-Cigarette Use (*n* = 502)	*p*-Value ^b^
Characteristic	*n* (%) ^a^	*n* (%) ^a^	*n* (%) ^a^	
**Sex**				**<0.001**
Male	1438 (55.4)	1124 (53.2)	314 (64.9)	
Female	1099 (44.6)	911 (46.8)	188 (35.1)	
**Race/Ethnicity**				**0.002**
Non-Hispanic White	1724 (66.8)	1359 (64.7)	365 (75.4)	
Non-Hispanic Black	239 (11.0)	207 (12.1)	32 (6.7)	
Hispanic	341 (12.9)	281 (13.0)	60 (12.2)	
Non-Hispanic Other/Multiracial	233 (9.3)	188 (10.2)	45 (5.7)	
**Education Level**				**0.006**
High school graduate or less	1159 (44.6)	897 (43.2)	262 (50.8)	
Some level of college education	900 (35.8)	718 (35.6)	182 (36.7)	
College graduate	478 (19.6)	420 (21.2)	58 (12.5)	
**Household Income Level**				0.100
<USD 25,000	380 (14.6)	298 (14.7)	82 (14.3)	
USD 25,000–USD 49,999	625 (24.3)	496 (23.4)	129 (28.1)	
≥USD 50,000	828 (35.3)	650 (34.7)	178 (37.6)	
Unspecified ^c^	704 (25.8)	591 (27.2)	113 (20.0)	
**Current Other Tobacco Use ^d^**				**<0.001**
No	2286 (91.4)	1913 (94.9)	373 (76.5)	
Yes	251 (8.6)	122 (5.1)	129 (23.5)	

*Notes:* Abbreviations: BRFSS, Behavioral Risk Factor Surveillance System; e-cigarette, electronic cigarette. ^a^ *n* refers to unweighted sample counts and weighted column percentages. ^b^ Bold font indicates statistical significance *p* < 0.05. ^c^ Unspecified category includes unknown and not sure. ^d^ Current other tobacco use includes combustible cigarettes or smokeless tobacco products.

**Table 2:** Participant characteristics based on cumulative number of ACEs among U.S. young adults 18–24 years old, 2021 BRFSS.

	Cumulative ACE Score				
	0 ACEs (*n* = 602)	1 ACE (*n* = 543)	2 ACEs (*n* = 378)	3 ACEs (*n* = 228)	≥4 ACEs (*n* = 786)
Characteristic	*n* (%) ^a^	*n* (%) ^a^	*n* (%) ^a^	*n* (%) ^a^	*n* (%) ^a^
**Current E-Cigarette Use**					
No	535 (90.4)	464 (84.2)	307 (81.1)	178 (78.1)	551 (71.8)
Yes	67 (9.6)	79 (15.8)	71 (18.9)	50 (21.9)	235 (28.2)
**Sex**					
Male	367 (56.2)	339 (61.8)	217 (53.3)	148 (59.6)	367 (49.8)
Female	235 (43.8)	204 (38.2)	161 (46.7)	80 (40.4)	419 (50.2)
**Race/Ethnicity**					
Non-Hispanic White	411 (69.0)	371 (64.0)	267 (69.8)	149 (65.7)	526 (66.0)
Non-Hispanic Black	52 (9.1)	67 (13.8)	36 (10.3)	22 (11.8)	62 (10.6)
Hispanic	87 (13.2)	61 (12.2)	47 (12.1)	40 (15.2)	106 (12.7)
Non-Hispanic Other/Multiracial	52 (8.7)	44 (10.0)	28 (7.8)	17 (7.3)	92 (10.7)
**Education Level**					
High school graduate or less	255 (38.5)	222 (43.7)	172 (42.1)	97 (41.0)	413 (52.4)
Some level of college education	200 (33.7)	205 (36.0)	138 (40.1)	86 (38.3)	217 (34.6)
College graduate	147 (27.8)	116 (20.3)	68 (17.8)	45 (20.7)	102 (13.0)
**Household Income Level**					
<USD 25,000	60 (9.1)	81 (16.0)	40 (9.8)	41 (20.9)	158 (17.6)
USD 25,000–USD 49,999	113 (19.2)	115 (17.7)	101 (24.7)	68 (30.7)	228 (30.8)
≥USD 50,000	224 (41.3)	193 (38.1)	129 (36.1)	65 (29.3)	217 (30.2)
Unspecified ^b^	205 (30.4)	154 (28.2)	108 (29.4)	54 (19.1)	183 (21.4)
**Current Other Tobacco Use ^c^**					
No	563 (96.5)	511 (93.6)	344 (90.9)	212 (93.5)	656 (85.3)
Yes	39 (3.5)	32 (6.4)	34 (9.1)	16 (6.5)	130 (14.7)

*Notes:* Abbreviations: ACEs, adverse childhood experiences; BRFSS, Behavioral Risk Factor Surveillance System; e-cigarette, electronic cigarette. ^a^ *n* refers to unweighted sample counts and weighted column percentages. ^b^ Unspecified category includes unknown and not sure. ^c^ Current other tobacco use is defined as current use of combustible cigarettes or smokeless tobacco products.

**Table 3:** Unadjusted and adjusted logistic regression model results of the association between cumulative number of ACEs and current e-cigarette use among U.S. young adults 18–24 years old, 2021 BRFSS.

	Current E-Cigarette Use			
	Unadjusted Logistic Regression Model		Adjusted Logistic Regression Model	
	OR (95%CI)	*p*-Value ^a^	AOR (95%CI)	*p*-Value ^b^
**Cumulative ACE Score**				
0 ACEs	**Ref**	**Ref**	Ref	Ref
1 ACE	**1.76 (1.01–3.07)**	**0.046**	1.25 (0.70–2.21)	0.450
2 ACEs	**2.18 (1.24–3.83)**	**0.007**	1.56 (0.85–2.84)	0.152
3 ACEs	**2.63 (1.41–4.90)**	**0.002**	**2.20 (1.15–4.23)**	**0.018**
≥4 ACEs	**3.69 (2.23–6.09)**	**<0.001**	**2.73 (1.58–4.71)**	**<0.001**
**Sex**				
Male	-	-	Ref	Ref
Female	-	-	**0.65 (0.48–0.89)**	**0.007**
**Race/Ethnicity**				
Non-Hispanic White	-	-	Ref	Ref
Non-Hispanic Black	-	-	**0.43 (0.25–0.72)**	**0.002**
Hispanic	-	-	**0.46 (0.26–0.81)**	**0.007**
Non-Hispanic Other/Multiracial	-	-	0.71 (0.44–1.13)	0.145
**Education Level**				
High school graduate or less	-	-	Ref	Ref
Some level of college education	-	-	1.03 (0.72–1.47)	0.881
College graduate	-	-	**0.57 (0.35–0.94)**	**0.026**
**Household Income Level**				
<USD 25,000	-	-	Ref	Ref
USD 25,000–USD 49,999	-	-	1.18 (0.74–1.89)	0.495
≥USD 50,000	-	-	1.21 (0.75–1.97)	0.436
Unspecified ^c^	-	-	0.82 (0.52–1.30)	0.408
**Current Other Tobacco Use ^d^**				
No	-	-	Ref	Ref
Yes	-	-	**3.61 (2.36–5.53)**	**<0.001**

*Notes:* Abbreviations: ACEs, adverse childhood experiences; e-cigarette, electronic cigarette; BRFSS, Behavioral Risk Factor Surveillance System; OR, odds ratio; CI, confidence interval; AOR, adjusted odds ratio; Ref, reference category. ^a^ Unadjusted logistic regression model results with no current e-cigarette use as the reference category. Bold font indicates statistical significance *p* < 0.05. ^b^ Adjusted logistic regression model results with no current e-cigarette use as the reference category and adjusting for participants’ sex, race/ethnicity, education level, household income level, and current other tobacco use. Bold font indicates statistical significance *p* < 0.05. ^c^ Unspecified category includes unknown and not sure. ^d^ Current other tobacco use is defined as current use of combustible cigarettes or smokeless tobacco products.

**Table 4:** Unadjusted and adjusted logistic regression model results of the associations between individual types of ACEs and current e-cigarette use among U.S. young adults 18–24 years old, 2021 BRFSS.

		Current E-Cigarette Use			
	Overall	Unadjusted Logistic Regression		Adjusted Logistic Regression	
Individual Type of ACE	*n* (%) ^a^	OR (95%CI)	*p*-Value ^b^	AOR (95%CI)	*p*-Value ^c^
**Child Emotionally Abused by Parent**					
No	196 (13.6)	Ref	Ref	Ref	Ref
Yes	306 (25.2)	**2.14 (1.60–2.87)**	**<0.001**	**1.83 (1.33–2.51)**	**<0.001**
**Parents Divorced**					
No	234 (17.4)	Ref	Ref	Ref	Ref
Yes	268 (21.5)	1.30 (0.98–1.73)	0.074	1.35 (0.98–1.88)	0.069
**Household Member with Mental Illness**					
No	258 (15.3)	Ref	Ref	Ref	Ref
Yes	244 (26.4)	**1.99 (1.50–2.65)**	**<0.001**	**1.91 (1.38–2.66)**	**<0.001**
**Household Member with Alcohol Problem**					
No	306 (16.0)	Ref	Ref	Ref	Ref
Yes	196 (27.5)	**2.00 (1.46–2.73)**	**<0.001**	**1.80 (1.27–2.55)**	**<0.001**
**Household Member used Illicit Drugs**					
No	353 (16.8)	Ref	Ref	Ref	Ref
Yes	149 (29.7)	**2.10 (1.52–2.89)**	**<0.001**	**1.69 (1.19–2.39)**	**0.003**
**Child Physically Abused by Parent**					
No	325 (16.4)	Ref	Ref	Ref	Ref
Yes	177 (28.1)	**1.99 (1.46–2.71)**	**<0.001**	**1.73 (1.21–2.46)**	**0.003**
**Household Members Engaged in Physical Violence**					
No	360 (17.3)	Ref	Ref	Ref	Ref
Yes	142 (27.1)	**1.77 (1.30–2.41)**	**<0.001**	**1.67 (1.18–2.34)**	**0.003**
**Household Member was Incarcerated**					
No	367 (17.8)	Ref	Ref	Ref	Ref
Yes	135 (25.9)	**1.62 (1.18–2.23)**	**0.003**	**1.31 (0.91–1.89)**	**0.150**
**Child Sexually Abused**					
No	398 (18.2)	Ref	Ref	Ref	Ref
Yes	104 (26.0)	**1.58 (1.09–2.30)**	**0.016**	**1.59 (1.01–2.53)**	**0.049**

*Notes:* Abbreviations: ACEs, adverse childhood experiences; e-cigarette, electronic cigarette; BRFSS, Behavioral Risk Factor Surveillance System; OR, odds ratio; CI, confidence interval; AOR, adjusted odds ratio; Ref, reference category. ^a^ *n* refers to unweighted sample counts and weighted row percentages. ^b^ Nine separate unadjusted logistic regression model results with no current e-cigarette use as the reference category. Bold font indicates statistical significance *p* < 0.05. ^c^ Nine separate adjusted logistic regression model results with no current e-cigarette use as the reference category. Bold font indicates statistical significance *p* < 0.05.

## Data Availability

The data that support the findings of this study are openly available at the CDC’s 2021 Behavioral Risk Factor Surveillance System website at https://www.cdc.gov/brfss/annual_data/annual_2021.html (accessed on 15 March 2023).
